# Complications of total hip and knee arthroplasty in solid organ transplant patients: a systematic review and meta-analysis

**DOI:** 10.1186/s42836-025-00343-w

**Published:** 2025-11-11

**Authors:** Dimitris Challoumas, Rohan Ramasubbu, Elliot Rooney, Angus Paterson, Almigdad Ali, Neal Millar, Bryn Jones

**Affiliations:** 1 School of Infection and Immunity, College of Medical, Veterinary and Life Sciences, University of Glasgow, Glasgow, G12 8TA UK; 2 Department of Trauma & Orthopaedic Surgery, Glasgow Royal Infirmary, Glasgow, G4 0SF UK

**Keywords:** Joint replacement, Renal transplant, Revision, Infection, Complications

## Abstract

**Background:**

This systematic review and meta-analysis aimed to quantify risks of complications associated with total hip and knee arthroplasty (THA, TKA) in patients with solid organ transplants (SOT) compared to the general population.

**Methods:**

The study was pre-registered on PROSPERO (CRD42023399043). Literature searches were performed looking for comparative studies reporting postoperative complication data of THA or TKA in patients with kidney, liver, pancreas, heart, or lung transplants versus controls. Outcomes of interest included incidence of blood transfusion, periprosthetic joint infection (PJI), periprosthetic fracture, deep venous thrombosis (DVT), pulmonary embolism (PE), mortality, hospital re-admission, and all-cause revision. The Newcastle Ottawa scale was used to assess study quality, and the GRADE for certainty of evidence.

**Results:**

A total of 13 studies participated in meta-analyses (10 in THA, 3 in TKA). Compared to controls, SOT patients (mixed data from all transplant types) had a significantly higher incidence of blood transfusion [THA OR 1.57 (1.36–1.80), TKA OR 1.37 (1.15–1.63)], PJI [THA OR 1.78 (1.01–3.12), TKA 3.11 (1.16–8.35)], DVT [THA OR 1.32 (1.04–1.66), TKA OR 1.56 (1.36–1.78)], and all-cause revision [only TKA OR 1.37 (1.15–1.63)]. THA in kidney transplant patients was associated with higher early mortality [OR 2.12 (1.38–3.25)] and 30-day re-admission [OR 1.62 (1.31–2.00)] compared to the general population. SOT was not associated with a higher incidence of post-operative PE after either THA or TKA [OR 0.94 (0.66–1.34), OR 0.89 (0.55–1.43), respectively]. The incidence of THA dislocation in mixed analyses with all SOT types was not statistically significant despite the unfavourable OR [1.62 (0.94–2.78)], but it was in the kidney and heart transplant subgroup analyses (OR 1.41 (1.16–1.73), OR 2.17 (1.47–3.20), respectively). The incidence of periprosthetic fracture was not higher in SOT patients compared to controls in those undergoing a THA [OR 1.07 (0.84–1.36)], but it was after a TKA [OR 1.79 (1.36–2.36)].

**Conclusion:**

THA and TKA are associated with an unfavourable complication profile in SOT patients compared to the general population. Decisions for or against arthroplasty surgery should be made on an individual basis with a multidisciplinary approach.

**Supplementary Information:**

The online version contains supplementary material available at 10.1186/s42836-025-00343-w.

## Introduction

The longevity of patients with solid organ transplants (SOT; kidney, liver, pancreas, heart, and lung) continues to increase, thanks to improvements in perioperative management and immunosuppressive treatment [1, 2]. In addition to the fact that SOT patients develop arthritis at similar rates to the general population, immunosuppressive regimens may lead to avascular necrosis—typically of the femoral head—estimated to occur in approximately 5% of these patients [3, 4]. Consequently, transplant recipients have a significantly greater likelihood of requiring joint replacement compared to the general population [5].

Previous literature has reported significant improvements in clinical outcomes in SOT patients undergoing total hip arthroplasty (THA) or total knee arthroplasty (TKA) [6]. However, it remains uncertain whether these benefits outweigh the associated risks, which are primarily driven by the medical complexity of this population and the requirement for lifelong immunosuppression. The decision to proceed with total joint arthroplasty in SOT patients should be made on a multidisciplinary basis—engaging all relevant medical professionals (surgeon, anaesthetist, internal medicine physician) and, importantly, the patient—after careful individualised assessment of the impact of arthritis on the patient’s quality of life.

Current data on complication profiles following THA and TKA in SOT patients are mostly derived from retrospective database reviews. Interpretation is challenging due to variation in reported outcomes, different transplant types, and the distinction between THA and TKA. This study aimed to systematically identify, summarise, and present the existing evidence and quantify the risks of complications, with the ultimate goal of informing healthcare professionals and patients and guiding the informed consent process.

## Materials and methods

This systematic review and meta-analysis was prospectively registered on PROSPERO (CRD42023399043) and conducted in accordance with PRISMA guidelines [7].

### Eligibility criteria

#### Types of studies

Observational studies with a control group were eligible for inclusion. Only studies published in English were considered.

#### Exposure—control

Studies of patients with any type of SOT undergoing primary THA or TKA were included if they also involved a matched control group without SOT. The following were excluded: (1) revision THA or TKA, (2) arthroplasty of joints other than the hip or knee, (3) partial hip (hemiarthroplasty) or knee (unicompartmental) arthroplasty, (4) studies reporting outcomes only for SOT patients without a matched control (i.e., case series), and (5) studies involving non-solid organ transplants (e.g., bone marrow).

#### Types of outcome measures

Blood transfusion, periprosthetic joint infection (PJI), dislocation (THA only), venous thromboembolism (deep vein thrombosis [DVT] and pulmonary embolism [PE]), mortality, and all-cause revision.

### Literature search

Search strategies included the following terms as keywords:

(solid organ transplant OR kidney transplant OR renal transplant OR liver transplant OR heart transplant OR lung transplant OR pancreas transplant) AND (arthroplasty OR joint replacement OR joint arthroplasty OR knee arthroplasty OR hip arthroplasty OR knee replacement OR hip replacement).

The databases Medline and Embase (via Ovid), Cochrane Central, and OpenGrey.eu were searched for both published and unpublished trials up to March 2025 by two authors. For unpublished or ongoing trials, the WHO International Clinical Trials Registry Platform, ClinicalTrials.gov, the EU Clinical Trials Register, and the ISRCTN registry were also searched. Reference lists of all eligible studies were manually screened. The search was repeated using the same terms as MeSH terms in Medline.

A total of 533 studies were identified after duplicates were removed. After excluding non-eligible study types, the remaining abstracts and full texts were screened independently by two authors. The PRISMA flow diagram is provided in the Supplementary Material.

### Data extraction

Data on patient and intervention characteristics, outcomes, and follow-up duration were extracted using a predesigned form in Microsoft Word version 16.43 (Microsoft Corp) by two authors independently. Where necessary, corresponding authors of studies published within the last 5 years were contacted for missing data.

#### Data handling—synthesis of results

Studies reporting the same outcome measures for SOT patients and matched controls were pooled in pairwise meta-analyses, provided there was no significant clinical heterogeneity (e.g., population, follow-up duration, intervention). Data were pooled separately for each SOT type (kidney, liver, heart, lung, pancreas) and for all SOT types combined. Separate analyses were conducted for THA and TKA; combined THA/TKA outcomes were not pooled, as no outcomes were reported by two or more such studies. Odds ratios (ORs) with 95% confidence intervals (CIs) were used for all outcomes.

### Risk of bias and certainty of evidence

The Newcastle–Ottawa Scale (NOS) was used to assess risk of bias (RoB) in cohort studies [8]. AHRQ thresholds (good, fair, poor quality) were applied. The GRADE tool was used to appraise certainty of evidence across five domains: RoB, imprecision, inconsistency, indirectness, and other confounding factors (including publication bias) [9]. Certainty started at “high” for diagnostic studies and was downgraded by one level for each concern. Certainty was upgraded if the effect size was large and statistically significant with narrow CIs, or downgraded for wide CIs. Single-study results began at “moderate” certainty. For combined SOT analyses, certainty was always downgraded one level for indirectness due to clinical heterogeneity.

All assessments were conducted independently by two authors, with discrepancies resolved by the senior author.

### Statistical analysis

Review Manager (RevMan) version 5 was used for pooled OR calculations, forest plots, and heterogeneity testing (Chi^2^ and I^2^). Publication bias was not assessed, as no meta-analysis included more than 10 studies. A random-effects model was used due to expected heterogeneity. Sensitivity analyses were conducted for meta-analyses with I^2^ > 75% and ≥ 3 studies; studies contributing to heterogeneity were removed and analyses re-run.

## Results

Seventeen studies met eligibility criteria and were included [1, 10–25]. Four were excluded from meta-analyses due to reporting mixed THA and TKA outcomes [1, 11, 12, 17]. Of the remaining 13 studies, 10 focused on THA and 3 on TKA (Table 1).

**Table 1:** Study characteristics table

First author et al. (year)	Procedure (THA vs TKA)	SOT Type (kidney, liver, etc.)	Population *n* = X, mean age = X in each group	Exposure vs Control matched for?	Outcomes assessed	Follow-up period for revision and PJI
Lim et al. (2012) [18]	THA	Kidney	Control group n = 72 with 94 THA (mean age 45 years) SOT group *n* = 30 patients with 45 THA (mean age 44 years)	Sex, BMI, age, AVN, Duration of follow-up	Pre and post op Harris Hip score Radiographic: wear, loosening, ectopic ossification Complications: Infection, dislocation, PPF, DVT, wound issues, nerve palsy, revision	Minimum 2-year follow-up Mean follow-up was 7.2 years for both groups
McCleery et al. (2010) [20]	TKA	Kidney	Control group n = 55,570 SOT group *n* = 25 (mean ages not stated)	Not matched	Early (< 90 days) and late infection (> 90 days) Early (< 365 days) and late > 365 days) all-cause revision	Not specified. Late revision was > 365 days
Agarwal et al. (2022) [10]	THA	Kidney, heart, lung, liver, pancreas	Control group *n* = 6196 (mean age 60–70 years) SOT group *n* = 3103 (mean age 60–70 years)	Age, gender, Charlson Comorbidity Index, obesity	PJI, DVT, PT, mortality, blood transfusion, all-cause revision	Minimum 2 years follow-up
Navale et al. (2017) [21]	THA	Kidney, heart, lung, liver, pancreas	Control group population 2,772,943 (mean age 60–70 years) SOT group *n* = 7558 (mean age 50–60 years)	Age, sex, race, insurance, hospital region, hospital location, and bed size, teaching hospital	PJI, dislocation, DVT, PT, blood transfusion	NA
Klement et al. (2016) [14]	TKA	Kidney, liver, lung, heart, and pancreas	Control group *n* = 1,685,295 (mean age 65–69 years) SOT group *n* = 3334 (mean age < 65 years)	Not matched	Medical and Surgical Outcomes at 30, 90, and “overall” Medical—MI, HF, Arrhythmia with and without AF, respiratory failure, DVT, PE, CVA, PNA, sepsis/SIRS, acute renal failure, anaemia, blood transfusion Surgical—MUA, Periproshetic infection, PPF, TKA revision, arthrotomy, patellar complications, extensor mechanism rupture	Minimum 2 years follow-up
Douglas et al. (2023) [13]	THA	Liver	Control group *n* = 10,246 (mean age 60 years) SOT group *n* = 513 (mean age 60 years)	Age, gender, Charlson Comorbidity Index, obesity, and diabetes	LOS, readmissions, blood transfusion, MI, DVT, PE, pneumonia, resp failure, dislocation, mechanical complications (PPF, aseptic loosening, Periprosthetic osteolysis)	90 days, 1 year, 2 years, and 5 years
Quinlan et al. (2022) [23]	THA	Heart, lung, kidney, pancreas, liver	Control group *n* = 4980 (mean age < 64 years) SOT group *n* = 996 (mean age < 64 years)	Age, gender, tobacco use, and Diabetes	Death, Hospital admission 30 and 90 d), ER visit (30 and 90 d), infection, all-cause revision, aseptic revision, dislocation	1 year
Malkani et al. (2020) [19]	THA	Kidney	SOT group *n* = 94 (mean age not reported) Control group *n* = 54,902 (mean age not reported)	Not matched	VTE, PJI, Dislocation, readmission, mortality. All were over 90 d, 0.5y, 1y, 2y & 5y	5 years
Tornero et al. (2015) [24]	THA	Kidney	SOT group *n* = 15 (mean age 53.8 years) Control group *n* = 74 (mean age 68.0 years)	Not matched	Blood transfusion, LOS, early postoperative complications (early PJI, dislocation, systemic infections, DVT & other complications), rate of revision (aseptic loosening, PJI or fracture), and overall prosthesis survival (1, 2, 5 & 10 years)	10y
Klika et al. (2015) [16]	TKA	SOT (not specified)	Control group *n* = 5,864,317 (mean age 66.8 years) SOT group *n* = 6,104 (mean age 60.8 years)	Patient characteristics (sex, age, race, household income, primary payer source), Hospital characteristics (region, bed size, location, surgeon volume, hospital volume), and comorbidities	LOS, costs per admission, and perioperative complications and adverse events [Medical complications, need for blood transfusion and surgical complications (either related to implant, eg, dislocation and fractures, or general, eg, wound-related, DVT/PE, …)]	not specified -infection rates are limited to the perioperative hospital stay until discharge
Klement et al. (2017) [15]	THA	Kidney, liver, lung, heart & pancreas	Control group *n* = 771,498 (> 65% 65–79y age range) SOT group *n* = 3,180 [Kidney *n* = 2,321, Liver *n* = 561, Lung *n* = 196, Heart *n* = 428, Pancreas *n* = 149] (> 60% were less than 60yrs of age) No mean age was provided	Not matched	A list of medical complications by 30 days post op, and surgical complications (PJI, dislocation, fracture, osteolysis + poly wear, mechanical complications, revision, and arthrotomy/I&D	Minimum 2 years
Oya et al. (2021) [22]	THA	Liver	Control group *n* = 27 (mean age 55.8 years) SOT group *n* = 7 with 9 THA (mean age 58.4 years)	Not matched	FBC, UE’s, LFT’s, operative time, intraoperative blood loss, incidence of perioperative complications, and the Harris Hip Score (HHS)	Mean 46.3 months (Range 24–60 months)
Varatharaj 2022 [25]	THA	Not listed	Control group *n* = 367,894 (mean age 66 years) SOT group *n* = 813 (mean age 61 years)	Not matched	Medical and Surgical Outcomes (elective procedure, dislocation, mortality/death, hematoma, wound disruption, infection, anemia, acute renal failure, hypotension, pulmonary embolism, myocardial infarction, pneumonia, blood transfusions, intra-operative complications, deep venous thrombosis, stroke, peri-prosthetic fractures, prosthetic hip dislocations, prosthetic loosening, length of stay, and health care-related expenditure)	Not reported

Follow-up ranged from 1 to 10 years, with 4 studies having ≥ 2 years follow-up. SOT-control matching was performed in 7 studies. Most did not report perioperative immunosuppressive therapy, assumed to vary widely both within and between studies. Reporting on implant types and cement use was also limited, contributing to presumed high heterogeneity.

Fifteen studies were rated as “good” quality and two as “poor” (Table 2). No results were downgraded for RoB due to this.

**Table 2:** Study quality assessment with the Newcastle–Ottawa tool

	**Selection**				**Comparability**	**Outcome**			**Overall**
Study	**1**	**2**	**3**	**4**	**1**	**1**	**2**	**3**	
Lim 2012 [18]	**✓**	**✓**	**✓**	**✓**	**✓ ✓**	✘	**✓**	**✓**	Good quality
McCleery et al. (2010) [20]	✘	✘	**✓**	**✓**	✘✘	✘	✘	✘	Poor quality
Agarwal 2022 [10]	**✓**	**✓**	**✓**	**✓**	**✓ ✓**	**✓**	**✓**	**✓**	Good quality
Navale 2017 [21]	**✓**	**✓**	**✓**	**✓**	**✓ ✓**	**✓**	✘	**✓**	Good quality
Klement 2016 [14]	**✓**	**✓**	**✓**	**✓**	✘ **✓**	**✓**	**✓**	✘	Good quality
Ahmed 2023 [11]	**✓**	**✓**	**✓**	**✓**	**✓ ✓**	**✓**	**✓**	**✓**	Good quality
Douglas 2023 [13]	**✓**	**✓**	**✓**	**✓**	**✓ ✓**	**✓**	**✓**	✘	Good Quality
Quinlan 2022 [23]	**✓**	**✓**	**✓**	**✓**	**✓ ✓**	**✓**	✘	**✓**	Good Quality
Malkani et al. (2020) [19]	**✓**	**✓**	**✓**	**✓**	✘**✓**	**✓**	**✓**	✘	Good quality
Tornero et al. (2015) [24]	**✓**	**✓**	**✓**	**✓**	✘**✓**	**✓**	**✓**	✘	Good quality
Klika et al. (2015) [16]	**✓**	**✓**	**✓**	**✓**	**✓✓**	**✓**	✘	**✓**	Good quality
Klement et al. (2017) [15]	**✓**	**✓**	**✓**	**✓**	✘**✓**	**✓**	**✓**	**✓**	Good quality
Kuo 2018 [17]	**✓**	**✓**	**✓**	**✓**	**✓ ✓**	**✓**	**✓**	**✓**	Good quality
Brown 2020 [1]	**✓**	**✓**	**✓**	**✓**	**✓ ✓**	**✓**	**✓**	**✓**	Good quality
Oya 2021 [22]	✘	**✓**	**✓**	**✓**	**✓** ✘	**✓**	**✓**	✘	Good quality
Varatharaj 2022 [25]	**✓**	**✓**	**✓**	**✓**	✘✘	**✓**	**✓**	**✓**	Poor quality
Dodin 2022 [12]	**✓**	**✓**	**✓**	**✓**	**✓** ✘	**✓**	**✓**	**✓**	Good quality

Table 3 summarises results for each outcome and the pooled analyses with GRADE certainty. Below, we present statistically significant meta-analysis results for all SOT types and specific subtypes. Sensitivity analyses and additional results are available in Table 3.

**Table 3:** Main results. Statistical significance is denoted by confidence intervals that do not include “1”

	**Transplant type**	**First author (year)**	**PJI**	**PP#**	**30 day re-admission**	**Dislocation (THA only)**	**DVT**	**PE**	**Mortality**	**Blood transfusion**	**All cause revision**	**Notes**
**THA only**	**Kidney**	Number of studies (total n SOT/Control)	4 (2478/826572)	2 (2384/771670)	-	4 (2478/826572)	2 (2384/771670)	1 (2321/771498)	1 (8669/54934)	2 (2339/771574)	2 (2346/1740865)	
		**Mean OR (95% CI)**	**1.50 (0.65, 3.49)**	**1.09 (0.81, 1.48)**	**-**	**1.41 (1.16, 1.73)**	**1.60 (1.30, 1.97)**	**0.90 (0.57, 1.43)**	**2.8 (1.8, 4.2)**	**2.39 (0.63, 9.00)**	**1.30 (1.05, 1.61)**	
		I^2^	32%	0%	-	0%	0%	-	-	83%	0%	
		**Certainty of Evidence**	**Moderate** ⊕ ⊕ ⊕ ∅	**High** ⊕ ⊕ ⊕ ⊕	**-**	**High** ⊕ ⊕ ⊕ ⊕	**High** ⊕ ⊕ ⊕ ⊕	**Moderate** ⊕ ⊕ ⊕ ∅	**Moderate** ⊕ ⊕ ⊕ ∅	**Low** ⊕ ⊕ ∅∅	**High** ⊕ ⊕ ⊕ ⊕	
	**Liver**	Number of studies (total n SOT/Control)	2 (1074/19961)	2 (570/771525)	-	2 (1083/781771)	2 (1083/781771)	3 (1083/781771)	-	2 (1074/781744)	2 (1074/781744)	
		**Mean OR (95% CI)**	**1.41 (0.94, 2.12)**	**0.54 (0.23, 1.27)**	**-**	**1.09 (0.76, 1.56)**	**1.25 (0.76, 2.05)**	**0.77 (0.39, 1.52)**	**-**	**1.51 (1.28, 1.77)**	**0.72 (0.48, 1.07)**	
		I^2^	14%	0%	-	0%	43%	0%	-	0%	0%	
		**Certainty of Evidence**	**Moderate** ⊕ ⊕ ⊕ ∅	**Moderate** ⊕ ⊕ ⊕ ∅	**-**	**Moderate** ⊕ ⊕ ⊕ ∅	**Moderate** ⊕ ⊕ ⊕ ∅	**Moderate** ⊕ ⊕ ⊕ ∅	**-**	**High** ⊕ ⊕ ⊕ ⊕	**High** ⊕ ⊕ ⊕ ⊕	
	**Lung**	Number of studies (total n SOT/Control)	1 (196/771498)	1 (196/771498)	-	1 (196/771498)	1 (196/771498)	1 (196/771498)	-	1 (196/771498)	1 (196/771498)	
		**Mean OR (95% CI)**	**0.99 (0.41, 2.41)**	**1.52 (0.63, 3.70)**	**-**	**0.84 (0.35, 2.05)**	**1.03 (0.42, 2.49)**	**3.02 (1.24, 7.34)**	**-**	**1.71 (1.29, 2.26)**	**0.55 (0.22, 1.33)**	
		I^2^	-	-		-	-	-	-	-	-	
		**Certainty of Evidence**	**Low** ⊕ ⊕ ∅∅	**Low** ⊕ ⊕ ∅∅	**-**	**Low** ⊕ ⊕ ∅∅	**Low** ⊕ ⊕ ∅∅	**Moderate** ⊕ ⊕ ⊕ ∅	**-**	**Moderate** ⊕ ⊕ ⊕ ∅	**Low** ⊕ ⊕ ∅∅	
	**Heart**	Number of studies (total n SOT/Control)	1 (428/771498)	1 (428/771498)	-	1 (428/771498)	1 (428/771498)	1 (428/771498)	-	1 (428/771498)	1 (428/771498)	
		**Mean OR (95% CI)**	**1.86 (1.19, 2.91)**	**0.69 (0.28, 1.66)**	**-**	**2.17 (1.47, 3.20)**	**2.33 (1.54, 3.51)**	**1.36 (0.56, 3.29)**	**-**	**1.25 (1.03, 1.52)**	**1.13 (0.74, 1.74)**	
		I^2^	-	-	-	-	-	-	-	-	-	
		**Certainty of Evidence**	**Moderate** ⊕ ⊕ ⊕ ∅	**Low** ⊕ ⊕ ∅∅	**-**	**Moderate** ⊕ ⊕ ⊕ ∅	**Moderate** ⊕ ⊕ ⊕ ∅	**Low** ⊕ ⊕ ∅∅	**-**	**Moderate** ⊕ ⊕ ⊕ ∅	**Low** ⊕ ⊕ ∅∅	
	**Pancreas**	Number of studies (total n SOT/Control)	1 (149/771498)	1 (149/771492)	-	1 (149/771492)	1 (149/771492)	1 (149/771498)	-	1 (149/771492)	1 (149/771492)	
		**Mean OR (95% CI)**	**1.32 (0.54, 3.21)**	**2.02 (0.83, 4.93)**	**-**	**1.12 (0.46, 2.73)**	**1.36 (0.56, 3.32)**	**0.39 (0.02, 6.19)**	**-**	**1.02 (0.72, 1.43)**	**0.73 (0.30, 1.77)**	
		I^2^	-	-	-	-	-	-	-	-	-	
		**Certainty of Evidence**	**Low** ⊕ ⊕ ∅∅	**Low** ⊕ ⊕ ∅∅	**-**	**Low** ⊕ ⊕ ∅∅	**Low** ⊕ ⊕ ∅∅	**Low** ⊕ ⊕ ∅∅	**-**	**Low** ⊕ ⊕ ∅∅	**Low** ⊕ ⊕ ∅∅	
	**All transplant types combined**	Number of studies (total n SOT/Control)	8 (16,320/2988831)	4 (4065/1139591)	2 (1509/15226)	8 (13,226/1492664)	7 (15,329/3928976)	6 (15,176/3928804)	2 (4912/379070)	6 (15,185/3928853)	6 (7436/837748)	
		**Mean OR (95% CI)**	**1.78 (1.01, 3.12)**	**1.07 (0.84, 1.36)**	**1.62 (1.31, 2.00)**	**1.62 (0.94, 2.78)**	**1.32 (1.04, 1.66)**	**0.94 (0.66, 1.34)**	**2.12 (1.38, 3.25)**	**1.57 (1.36, 1.80)**	**0.84 (0.58, 1.20)**	
		I^2^	93%	0%	0%	93%	41%	43%	0%	81%	68%	
		**Certainty of Evidence**	**Moderate** ⊕ ⊕ ⊕ ∅	**Moderate** ⊕ ⊕ ⊕ ∅	**Moderate** ⊕ ⊕ ⊕ ∅	**V. Low** ⊕ ∅∅∅	**Moderate** ⊕ ⊕ ⊕ ∅	**Moderate** ⊕ ⊕ ⊕ ∅	**Moderate** ⊕ ⊕ ⊕ ∅	**V. Low** ⊕ ∅∅∅	**Low** ⊕ ⊕ ∅∅	
		**Mean OR (95% CI) after sensitivity analyses**	**1.46 (0.94, 2.27)** I^2^ = 0% **Moderate** ⊕ ⊕ ⊕ ∅	**-**	**-**	**1.41 (1.22, 1.64)** I^2^ = 0% **Low** ⊕ ⊕ ∅∅	**-**	**-**	**-**	**1.43 (1.30, 1.58)** I^2^ = 69% **Low** ⊕ ⊕ ∅∅	**-**	
**TKA only**	**Kidney**	Number of studies (total n SOT/Control)	2 (2346/1740865)	1 (2321/1685295)	-	-	1 (2321/1685295)	1 (2321/1685295)		2 (6082/7549612)	1 (2321/1685295)	
		**Mean OR (95% CI)**	**3.18 (1.26, 7.99)**	**1.61 (1.14, 2.29)**	**-**	**-**	**1.57 (1.30, 1.89)**	**0.59 (0.38, 0.93)**		**1.42 (1.33, 1.51)**	**1.29 (1.04, 1.60)**	
		I^2^	68%	-	-	-	-	-		0%		
		**Certainty of Evidence**	**Moderate** ⊕ ⊕ ⊕ ∅	**Moderate** ⊕ ⊕ ⊕ ∅	**-**	**-**	**Moderate** ⊕ ⊕ ⊕ ∅	**Moderate** ⊕ ⊕ ⊕ ∅		**High** ⊕ ⊕ ⊕ ⊕	**Moderate** ⊕ ⊕ ⊕ ∅	
	**Liver**	Number of studies (total n SOT/Control)	1 (772/1685295)	1 (772/1685295)	-	-	1 (772/1685295)	1 (772/1685295)		2 (2149/7549612)	1 (772/1685295)	
		**Mean OR (95% CI)**	**2.31 (1.71, 3.11)**	**0.45 (0.03, 7.15)**	**-**	**-**	**1.58 (1.15, 2.19)**	**0.05 (0.00, 0.75)**		**1.76 (1.34, 2.31)**	**1.54 (1.09, 2.18)**	
		I^2^	-	-	-	-	-	-		87%		
		**Certainty of Evidence**	**Moderate** ⊕ ⊕ ⊕ ∅	**Low** ⊕ ⊕ ∅∅	**-**	**-**	**Low** ⊕ ⊕ ∅∅	**Low** ⊕ ⊕ ∅∅		**Moderate** ⊕ ⊕ ⊕ ∅	**Moderate** ⊕ ⊕ ⊕ ∅	
	**Lung**	Number of studies (total n SOT/Control)	1 (129/1685295)	1 (129/1685295)	-	-	1 (129/1685295)	1 (129/1685295)		1 (129/1685295)	1 (129/1685295)	
		**Mean OR (95% CI)**	**0.14 (0.01, 2.26)**	**0.45 (0.03, 7.15)**	**-**	**-**	**0.11 (0.01, 1.85)**	**0.28 (0.02, 4.47)**		**1.35 (0.93, 1.98)**	**0.13 (0.01, 2.08)**	
		I^2^	-	-	-		-	-		-		
		**Certainty of Evidence**	**Low** ⊕ ⊕ ∅∅	**Low** ⊕ ⊕ ∅∅	**-**		**Low** ⊕ ⊕ ∅∅	**Low** ⊕ ⊕ ∅∅		**Low** ⊕ ⊕ ∅∅	**Low** ⊕ ⊕ ∅∅	
	**Heart**	Number of studies (total n SOT/Control)	1 (412/1685295)	1 (412/1685295)	-	-	1 (412/1685295)	1 (412/1685295)		1 (412/1685295)	1 (412/1685295)	
		**Mean OR (95% CI)**	**2.16 (1.42, 3.28)**	**0.14 (0.01, 2.24)**	**-**	**-**	**1.68 (1.09, 2.58)**	**0.09 (0.01, 1.40)**		**1.19 (0.96, 1.49)**	**0.04 (0.00, 0.65)**	
		I^2^	-	-	-	-	-	-		-	-	
		**Certainty of Evidence**	**Moderate** ⊕ ⊕ ⊕ ∅	**Low** ⊕ ⊕ ∅∅	**-**	**-**	**Low** ⊕ ⊕ ∅∅	**Low** ⊕ ⊕ ∅∅		**Moderate** ⊕ ⊕ ⊕ ∅	**Low** ⊕ ⊕ ∅∅	
	**Pancreas**	Number of studies (total n SOT/Control)	1 (167/1685295)	1 (167/1685295)	-	-	1 (167/1685295)	1 (167/1685295)		1 (167/1685295)	1 (167/1685295)	
		**Mean OR (95% CI)**	**0.11 (0.01, 1.75)**	**2.12 (0.13, 34.05)**	**-**	**-**	**2.10 (1.14, 3.87)**	**0.21 (0.01, 3.45)**		**1.47 (1.06, 2.03)**	**0.10 (0.01, 1.60)**	
		I^2^	-		-	-	-	-			-	
		**Certainty of Evidence**	**Low** ⊕ ⊕ ∅∅	**Low** ⊕ ⊕ ∅∅	**-**	**-**	**Moderate** ⊕ ⊕ ⊕ ∅	**Low** ⊕ ⊕ ∅∅		**Moderate** ⊕ ⊕ ⊕ ∅	**Low** ⊕ ⊕ ∅∅	
	**All transplant types combined**	Number of studies (total n SOT/Control)	2 (3359/1740865)	1 (3334/1685295)	-	-	2 (9438/7549612)	2 (9438/7549612)		2 (9438/7549612)	2 (3359/1740865)	
		**Mean OR (95% CI)**	**3.11 (1.16, 8.35)**	**1.79 (1.36, 2.36)**	**-**	**-**	**1.56 (1.36, 1.78)**	**0.89 (0.55, 1.43)**		**1.53 (1.35, 1.74)**	**1.37 (1.15, 1.63)**	
		I^2^	72%	-	-	-	0%	73%		84%	0%	
		**Certainty of Evidence**	**Low** ⊕ ⊕ ∅∅	**Low** ⊕ ⊕ ∅∅	**-**	**-**	**Moderate** ⊕ ⊕ ⊕ ∅	**Low** ⊕ ⊕ ∅∅		**Low** ⊕ ⊕ ∅∅	**Moderate** ⊕ ⊕ ⊕ ∅	

### Total hip arthroplasty

The meta-analysis results of THA outcomes in SOT patients versus matched controls, with their forest plots, pooled ORs, and accompanying 95% CI and heterogeneity tests, can be seen in the Supplementary material.

#### Blood transfusion

Patients with a SOT (mixed data) had a significantly higher incidence of blood transfusion postoperatively than controls at statistical significance [OR 1.57 (1.36–1.80), very low certainty]. Liver, lung, and heart transplants were also associated with a higher incidence of blood transfusion, at high, moderate, and moderate certainty of evidence, respectively [OR 1.51 (1.28–1.77), OR 1.71 (1.29–2.26), OR 1.25 (1.03–1.52), respectively].

#### Periprosthetic joint infection

Those with a SOT (mixed data) had a higher incidence of PJI [OR 1.78 (1.01–3.12), moderate certainty]. Heart transplants were also associated with a higher incidence of PJI [OR 1.86 (1.19–2.91), moderate certainty of evidence]. There were no other significant differences between SOT and control in the other SOT subtypes.

#### Dislocation

Despite the high OR favouring the control group, the difference between the SOT group (mixed data) and controls was statistically non-significant [OR 1.62 (0.94–2.78), v. low certainty]. Significant differences were found in those with kidney and heart transplants, favouring the control groups [OR 1.41 (1.16–1.73), high certainty, and OR 2.17 (1.47–3.20), moderate certainty].

#### Periprosthetic fracture

Patients with SOT (mixed data) did not have a higher incidence of sustaining a periprosthetic fracture compared to controls [OR 1.07 (0.84–1.36), moderate certainty]. This was the case for all subtypes, too.

#### Venous thromboembolism

Patients with SOT (mixed data) had a significantly higher incidence of DVT but not PE compared to controls [OR 1.32 (1.04–1.66), OR 0.94 (0.66–1.34), respectively, both moderate certainty]. Kidney and heart transplants were associated with a higher incidence of DVT compared to controls [OR 1.60 (1.30–1.97), high certainty, OR 2.33 (1.54–3.51), moderate certainty, respectively], and lung transplants with a higher incidence of PE [OR 3.02 (1.24–7.34), moderate certainty].

#### Hospital re-admission

Patients with a SOT (mixed data) had a significantly higher incidence of being re-admitted to hospital within 30 days of their THA compared to controls [OR 1.62 (1.31–2.00), moderate certainty]. There were no data for SOT subtype analyses.

#### Mortality

Patients with a SOT (mixed data) had a higher incidence of early mortality (up to 90 days) compared to controls [OR 2.12 (1.38–3.25), moderate certainty]. Kidney transplant patients undergoing THA had a higher incidence of 5-year mortality compared to controls [OR 2.8 (1.8–4.2), moderate certainty].

#### All-cause revision

Patients with a SOT (mixed data) did not have a higher incidence of all-cause revision compared to controls [OR 0.84 (0.58–1.20), low certainty]. Among all subtypes, only kidney transplants were associated with a higher risk of all-cause revision [OR 1.30 (1.05–1.61), high certainty].

### Total knee arthroplasty

The meta-analysis results of TKA outcomes in SOT patients versus matched controls, with their forest plots, pooled ORs, and accompanying 95% CI and heterogeneity tests, can be seen in the Supplementary material.

#### Blood transfusion

Patients with a SOT (mixed data) had a significantly higher incidence of blood transfusion postoperatively than controls at statistical significance [OR 1.53 (1.35–1.74), moderate certainty]. Among SOT subtypes, kidney, liver, and pancreas transplants were also associated with a higher risk [OR 1.29 (1.04–1.60), moderate certainty; OR 1.54 (1.09–2.18), low certainty; OR 1.47 (1.06–2.03), low certainty].

#### Periprosthetic joint infection

Those with a SOT (mixed data) had a significantly higher incidence of PJI compared to controls [OR 3.11 (1.16–8.35), low certainty]. There were also significant differences in the kidney, liver, and heart subgroups [OR 3.18 (1.26–7.99), OR 2.31 (1.71–3.11), and OR 2.16 (1.42–3.28), respectively, all moderate certainty].

#### Periprosthetic fracture

Patients with a SOT (mixed data) had a significantly higher incidence of sustaining a periprosthetic fracture compared to controls [OR 1.79 (1.36–2.36), low certainty]. Among SOT subtypes, those with a kidney transplant were at higher risk of a periprosthetic fracture compared to controls [OR 1.61 (1.14–2.29), moderate certainty].

#### Venous thromboembolism

Patients with a SOT (mixed data) had a significantly higher incidence of DVT but not PE compared to controls [OR 1.56 (1.36–1.78), moderate certainty, and OR 0.89 (0.55–1.43), low certainty, respectively]. Kidney, liver, heart, and pancreas transplants were also associated with a higher incidence of DVT [OR 1.57 (1.30–1.89), moderate certainty; OR 1.58 (1.15–2.19), low certainty; OR 1.68 (1.09–2.58), low certainty, and OR 2.10 (1.14–3.87), moderate certainty, respectively]. No SOT subtypes were associated with a higher incidence of PE.

#### All-cause revision

There was a higher incidence of all-cause revision in those with a SOT (mixed data) compared to controls [OR 1.37 (1.15–1.63, moderate certainty]. A significantly higher incidence was also observed with kidney and liver transplants [OR 1.29 (1.04–1.60) and OR 1.54 (1.09, 2.18), respectively, both moderate certainty].

## Discussion

This study aimed to quantify perioperative complication risks in SOT patients undergoing THA or TKA, to better inform surgical decision-making and the consent process. We conducted pooled analyses for all SOTs and for each subtype. Most outcomes showed unfavourable ORs for SOT patients compared to controls, although the certainty of evidence varied (very low to high), as determined by the GRADE criteria. Results with low or very low certainty should be interpreted cautiously.

Our findings indicate a significantly increased incidence of blood transfusion, DVT, and PJI in SOT patients after THA or TKA. Additionally, early postoperative mortality, all-cause revision, and 30-day readmission rates were higher in kidney transplant recipients undergoing THA, and periprosthetic fractures and all-cause revision were more frequent in SOT patients undergoing TKA.

The results were mainly based on large database studies. For instance, Klika et al. studied 6,104 SOT patients vs. 5,870,421 controls and found higher complication rates, longer hospital stays, and increased blood transfusions [16]. Klement et al., in a cohort of 3,339 SOT patients vs. 1,685,295 controls, reported most complications occurring within 90 days postoperatively [14]. Subgroup analysis showed kidney transplant recipients had the highest complication rates, which aligns with other studies [26].

Functional outcomes after THA/TKA in SOT patients appear favourable, with improvements in pain, mobility, and quality of life [6, 27–29]. However, these benefits come at the expense of increased complication risks, longer hospital stays, and higher costs [1, 27, 30, 31]. Early mortality may also be elevated in SOT patients. Some studies showed no difference in mortality between transplant patients and controls [11, 12, 17], while others reported significantly higher long-term mortality (OR 7.42) in SOT patients [1].

Implant survival data in SOT patients undergoing total joint arthroplasty are limited but generally acceptable. For THA, Chalmers et al. reported 2-year and 5-year survival rates of 94% and 92% [32], and for TKA, Ledford et al. found 98% survival at 2 years and 93% at 5 years [33]; Wu et al. reported mixed survival of 96% at 1 year and 94% at 4 years for THA and TKA [34]. These are lower than survival rates in the general population, which are around 98.5% and 97.2% for THA and 97.6% and 96.1% for TKA at 5 and 10 years, respectively, per the National Joint Registry [35]. These findings support our results showing higher all-cause revision rates in SOT patients, highlighting the need for transparent patient counselling.

There have been several advancements in solid organ transplantation in recent years. These predominantly relate to improved patient and donor selection, perioperative care, and better immunosuppressant regimes with a smaller reliance on steroids [1]. Despite these, patients with SOTs have significant risk factors for complications after total joint arthroplasty compared to the general population, which are likely responsible for the unfavourable complication profile. These include immunosuppression (increased risk of infection), poor bone quality due to metabolic abnormalities and long-term steroid use (increased risk of periprosthetic fractures and implant loosening), anaemia and nutritional deficiencies (increased risk of blood transfusion, wound healing issues and mortality), poor soft tissue quality (may predispose to dislocations and wound healing problems) and general medical complexity, frailty and other co-morbidities.

Despite the higher complication rates, the improved clinical outcomes in SOT patients should not be overlooked. Surgical decisions must be made on an individual basis, considering frailty, pain, quality of life, baseline mobility, functional needs, and comorbidities. Collaboration among surgeons, anaesthetists, and physicians is essential, with an emphasis on thorough informed consent and preoperative optimisation.

Our study has limitations inherent to the included studies, which were all retrospective and had heterogeneous populations, interventions, and outcomes. Most did not use propensity score matching, introducing bias. Database studies also rely on accurate coding, which may affect validity.

## Conclusion

We found increased rates of blood transfusion, PJI, DVT, and all-cause revision in SOT patients undergoing THA or TKA, compared to the general population. These risks were quantified, and the certainty of evidence assessed. Surgical decisions should be made on an individualised, multidisciplinary basis, with comprehensive patient counselling regarding risks and benefits.

## Supplementary Information

Supplementary Material 1.

## Data Availability

The datasets supporting the conclusions of this article ar) included within the article (and its additional file(s)).
