# Supplementary material for: Complications of total hip and knee arthroplasty in solid organ transplant patients: a systematic review and meta-analysis
Source: Arthroplasty. 2025 Nov 11;7:56. doi: 10.1186/s42836-025-00343-w (PMC12604258; doi:10.1186/s42836-025-00343-w)
Supplement: Supplementary file 1 — Supplementary Material 1. [file 42836_2025_343_MOESM1_ESM.docx]

**Supplementary Figures**

**PRISMA flow diagram**

**Identification of studies via databases and registers**

Records removed *before screening*:

Duplicate records removed (n = 553)

Records removed for other reasons (n = 0)

Records identified from:

Databases (n = 1135)

**Identification**

Titles screened

(n = 582)

Records excluded

(n = 556)

Reports sought for retrieval

(n = 26)

Reports not retrieved

(n = 0)

**Screening**

Reports excluded:

Ineligible populations (n = 7)

Mixed THA/TKA populations without subgroup analyses for each (n=4)

Reviews (n = 1)

Ineligible statistical measures (n = 1)

Reports assessed for eligibility

(n = 26)

Studies found eligible for data collection

(n = 17)

**Included**

Studies included in meta-analyses

(n = 13)

**Forest Plots**


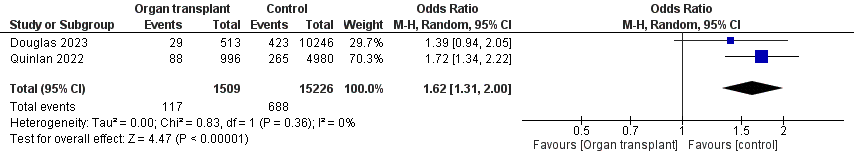


Solid organ transplant (all types) vs control, 30 day readmission, total hip arthroplasty


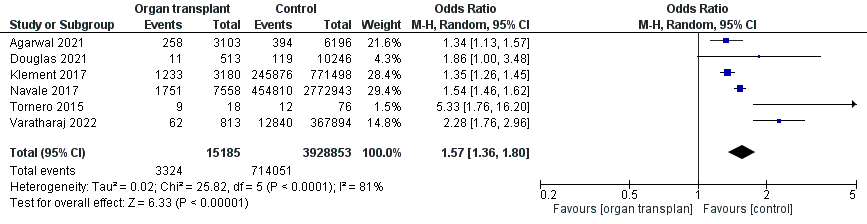


Solid organ transplant (all types) vs control, blood transfusion, total hip arthroplasty


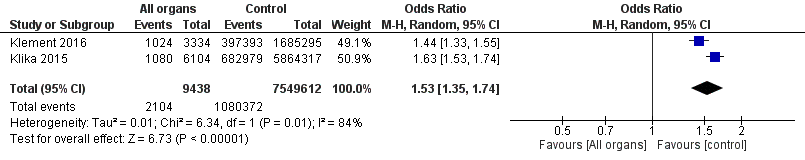


Solid organ transplant (all types) vs control, blood transfusion, total knee arthroplasty


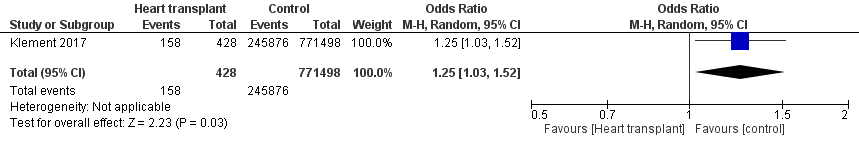


Solid organ transplant (heart) vs control, blood transfusion, total hip arthroplasty


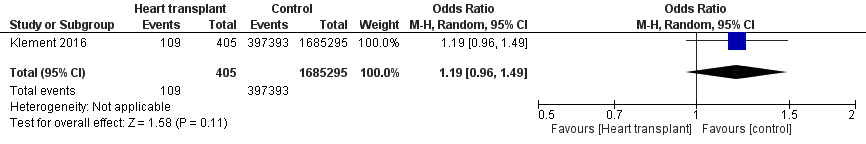


Solid organ transplant (heart) vs control, blood transfusion, total knee arthroplasty


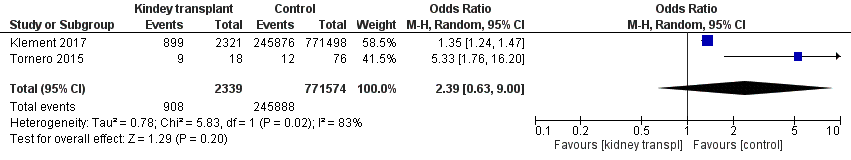


Solid organ transplant (kidney) vs control, blood transfusion, total hip arthroplasty


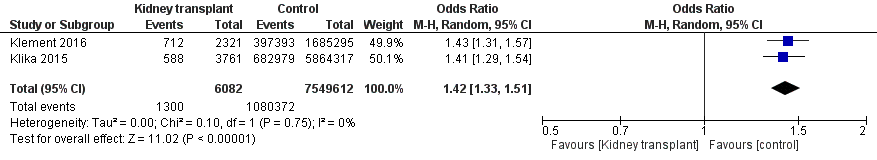


Solid organ transplant (kidney) vs control, blood transfusion, total knee arthroplasty


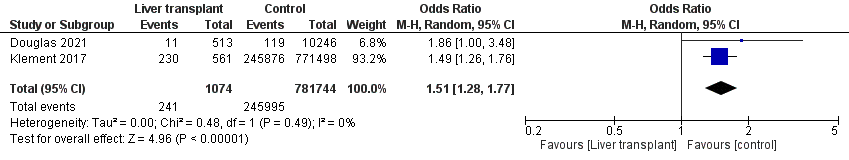


Solid organ transplant (liver) vs control, blood transfusion, total hip arthroplasty


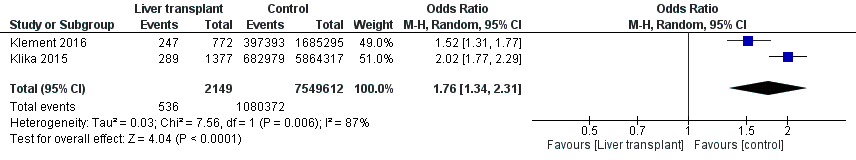


Solid organ transplant (liver) vs control, blood transfusion, total knee arthroplasty


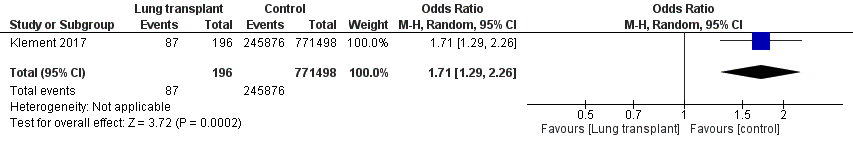


Solid organ transplant (lung) vs control, blood transfusion, total hip arthroplasty


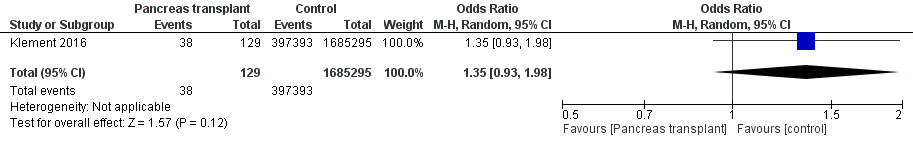


Solid organ transplant (lung) vs control, blood transfusion, total knee arthroplasty


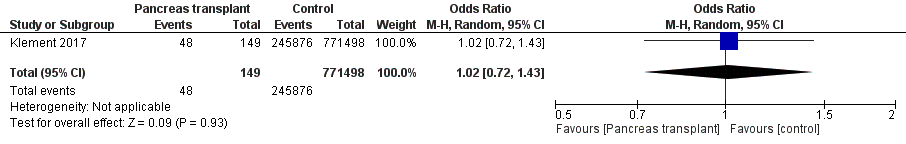


Solid organ transplant (pancreas) vs control, blood transfusion, total hip arthroplasty


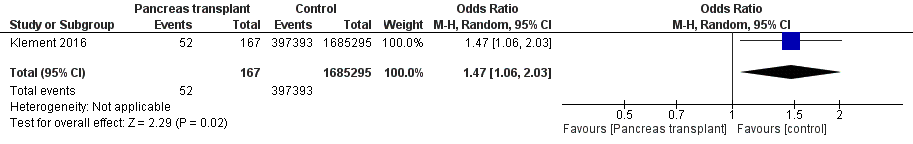


Solid organ transplant (pancreas) vs control, blood transfusion, total knee arthroplasty


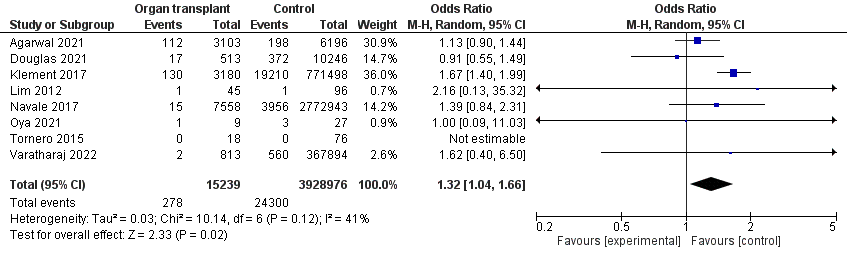


Solid organ transplant (all types) vs control, DVT, total hip arthroplasty


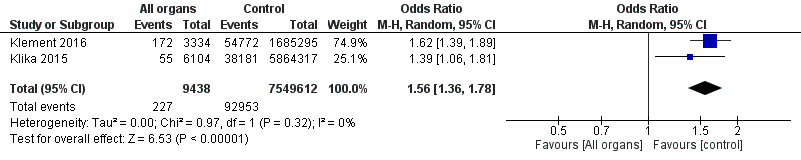


Solid organ transplant (all types) vs control, DVT, total knee arthroplasty


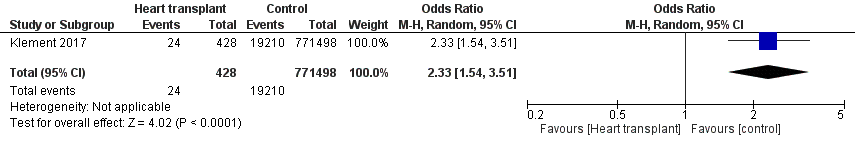


Solid organ transplant (heart) vs control, DVT, total hip arthroplasty


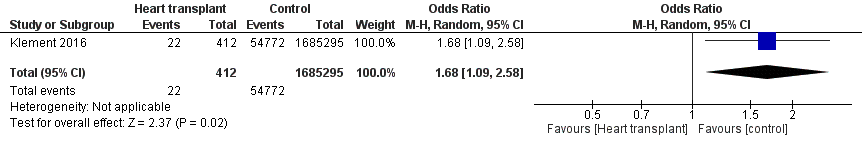


Solid organ transplant (heart) vs control, DVT, total knee arthroplasty


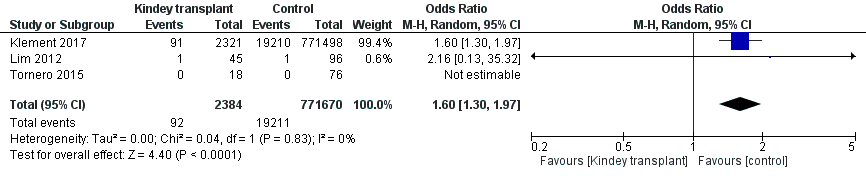


Solid organ transplant (kidney) vs control, DVT, total hip arthroplasty


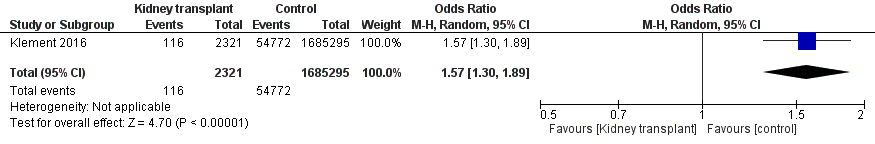


Solid organ transplant (kidney) vs control, DVT, total knee arthroplasty


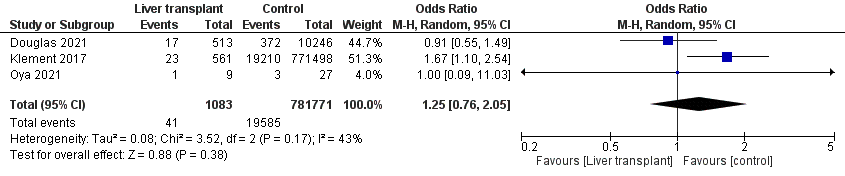


Solid organ transplant (liver) vs control, DVT, total hip arthroplasty


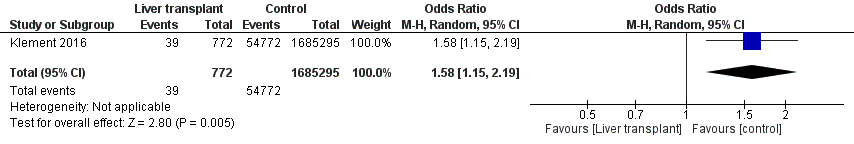


Solid organ transplant (liver) vs control, DVT, total knee arthroplasty


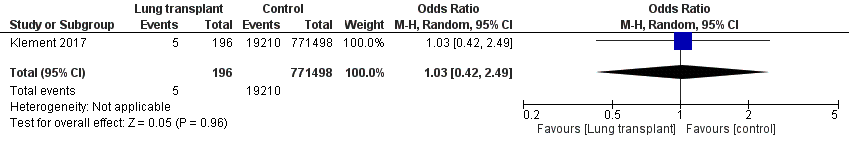


Solid organ transplant (lung) vs control, DVT, total hip arthroplasty


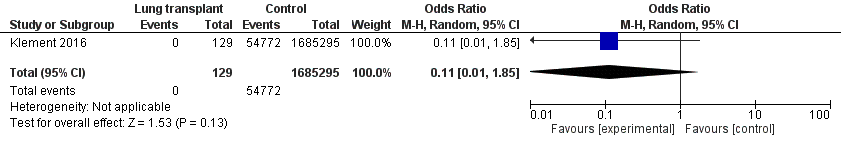


Solid organ transplant (lung) vs control, DVT, total knee arthroplasty


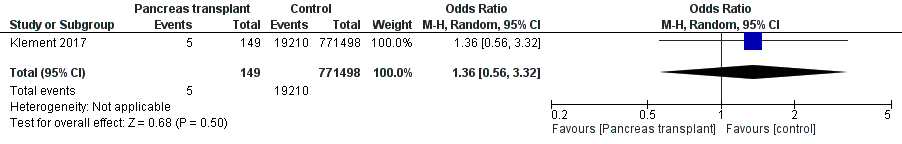


Solid organ transplant (pancreas) vs control, DVT, total hip arthroplasty


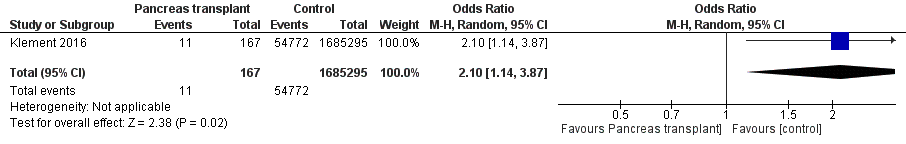


Solid organ transplant (pancreas) vs control, DVT, total knee arthroplasty


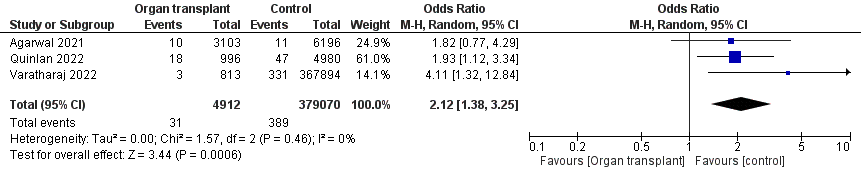


Solid organ transplant (all types) vs control, mortality, total hip arthroplasty


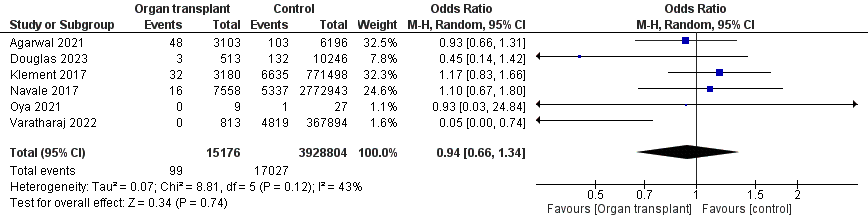


Solid organ transplant (all types) vs control, PE, total hip arthroplasty


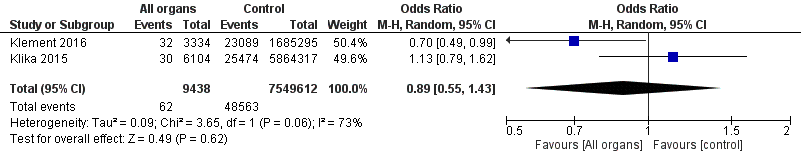


Solid organ transplant (all types) vs control, PE, total knee arthroplasty


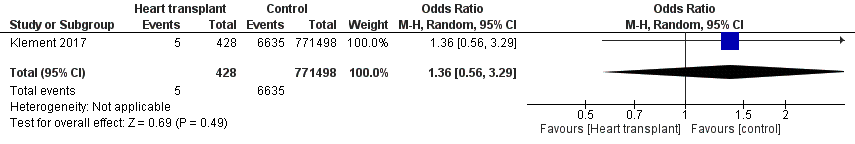


Solid organ transplant (heart) vs control, PE, total hip arthroplasty


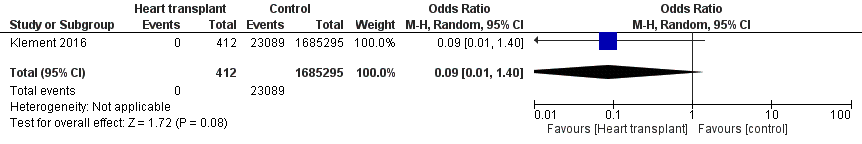


Solid organ transplant (heart) vs control, PE, total knee arthroplasty


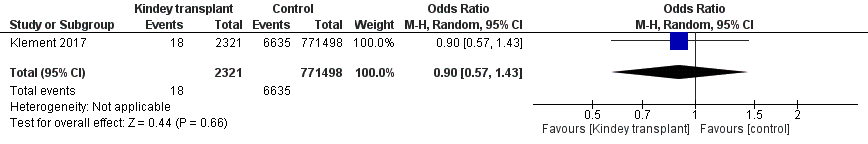


Solid organ transplant (kidney) vs control, PE, total hip arthroplasty


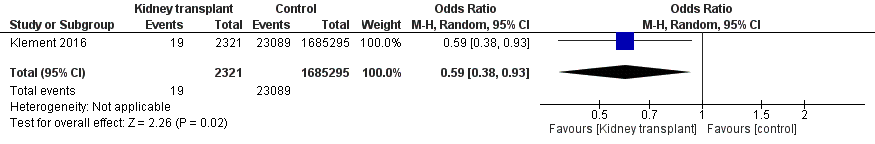


Solid organ transplant (kidney) vs control, PE, total knee arthroplasty


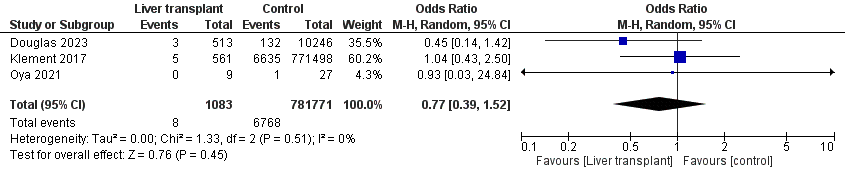


Solid organ transplant (liver) vs control, PE, total hip arthroplasty


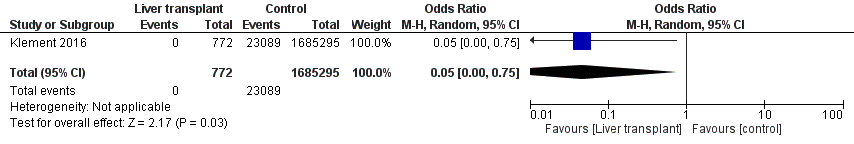


Solid organ transplant (liver) vs control, PE, total knee arthroplasty


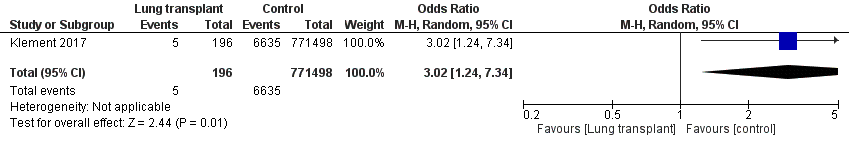


Solid organ transplant (lung) vs control, PE, total hip arthroplasty


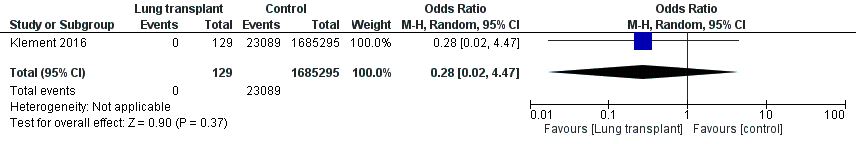


Solid organ transplant (lung) vs control, PE, total knee arthroplasty


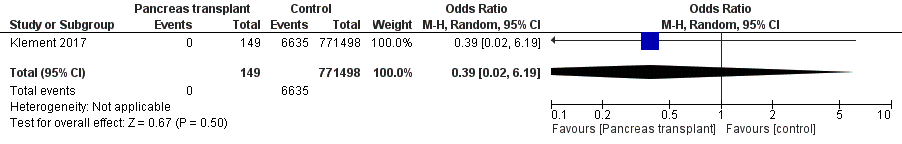


Solid organ transplant (pancreas) vs control, PE, total hip arthroplasty


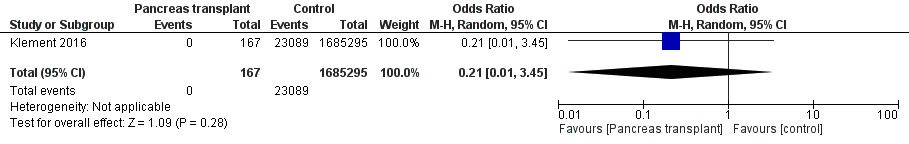


Solid organ transplant (pancreas) vs control, PE, total knee arthroplasty


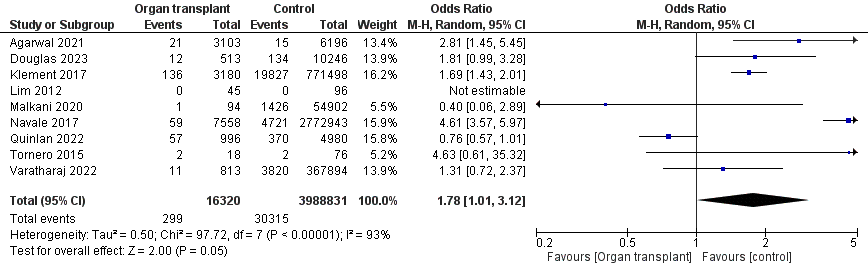


Solid organ transplant (all types) vs control, PJI, total hip arthroplasty


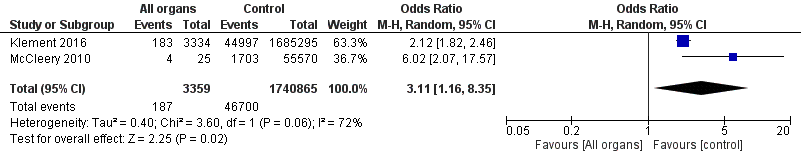


Solid organ transplant (all types) vs control, PJI, total knee arthroplasty


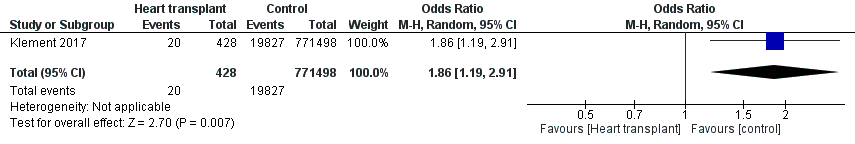


Solid organ transplant (heart) vs control, PJI, total hip arthroplasty


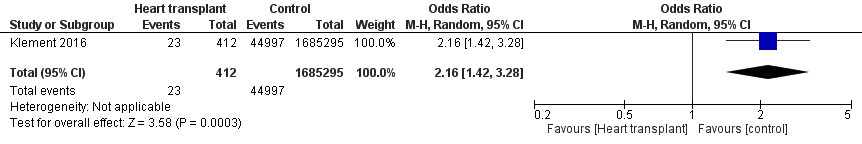


Solid organ transplant (heart) vs control, PJI, total knee arthroplasty


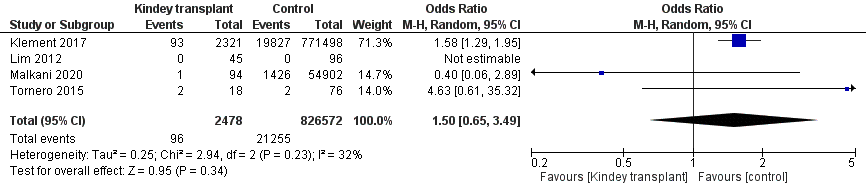


Solid organ transplant (kidney) vs control, PJI, total hip arthroplasty


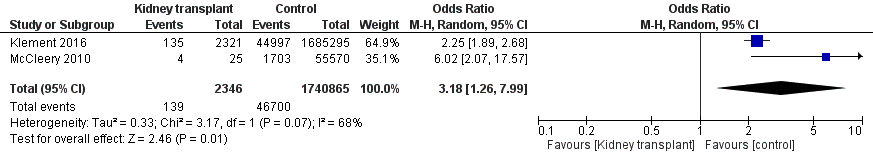


Solid organ transplant (kidney) vs control, PJI, total knee arthroplasty


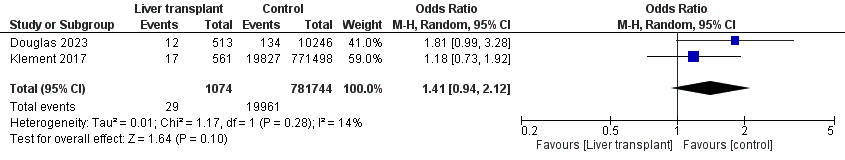


Solid organ transplant (liver) vs control, PJI, total hip arthroplasty


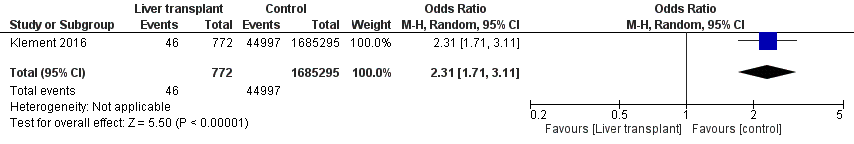


Solid organ transplant (liver) vs control, PJI, total knee arthroplasty


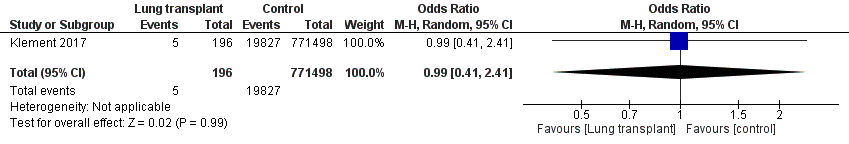


Solid organ transplant (lung) vs control, PJI, total hip arthroplasty


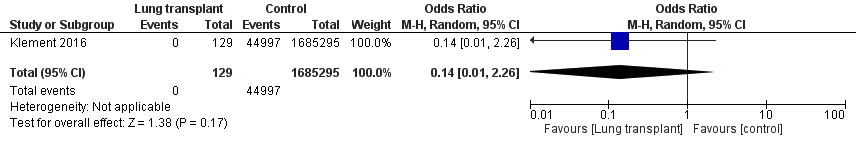


Solid organ transplant (lung) vs control, PJI, total knee arthroplasty


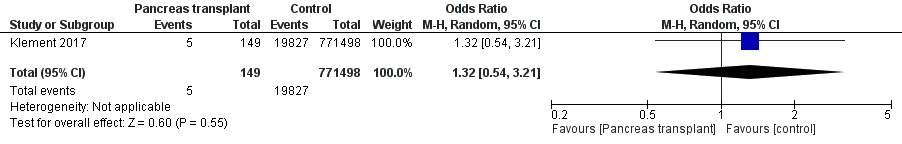


Solid organ transplant (pancreas) vs control, PJI, total hip arthroplasty


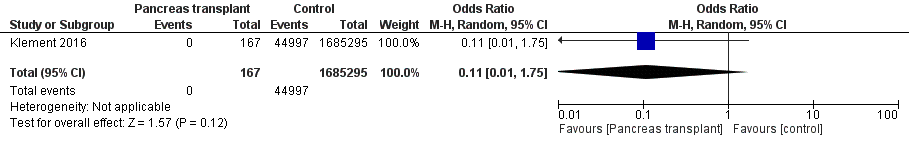


Solid organ transplant (pancreas) vs control, PJI, total knee arthroplasty


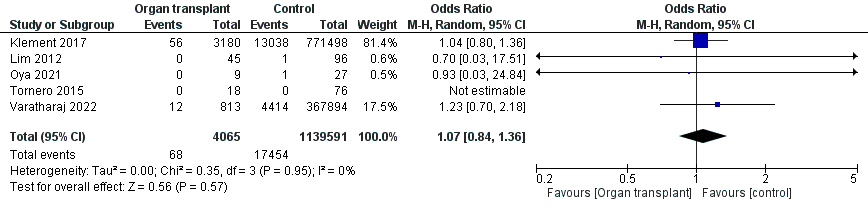


Solid organ transplant (all types) vs control, PP#, total hip arthroplasty


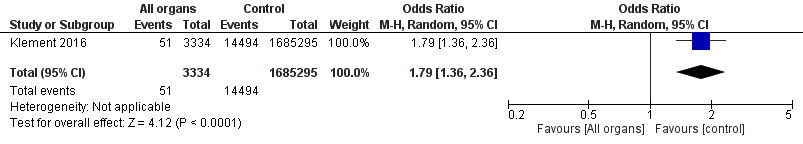


Solid organ transplant (all types) vs control, PP#, total knee arthroplasty


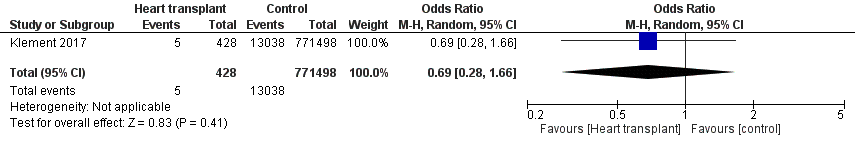


Solid organ transplant (heart) vs control, PP#, total hip arthroplasty


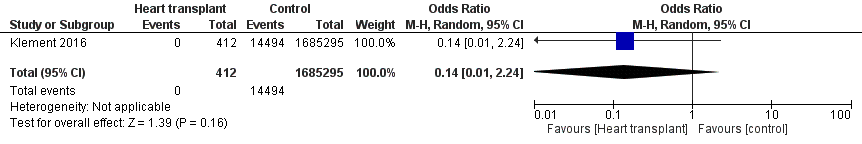


Solid organ transplant (heart) vs control, PP#, total knee arthroplasty


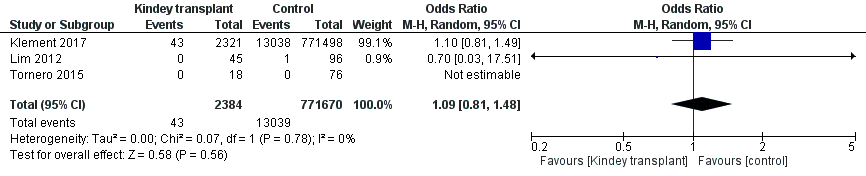


Solid organ transplant (kidney) vs control, PP#, total hip arthroplasty


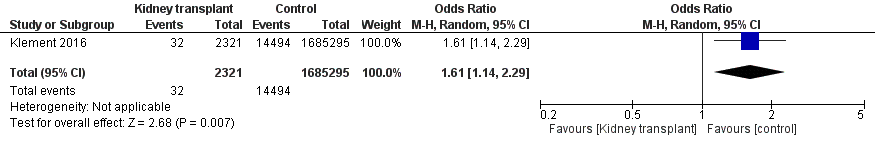


Solid organ transplant (kidney) vs control, PP#, total knee arthroplasty


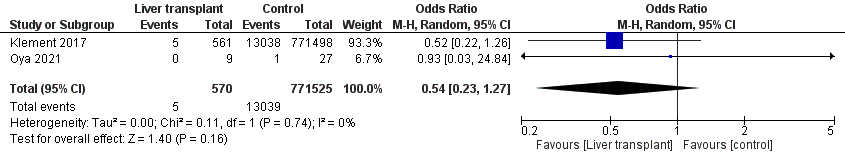


Solid organ transplant (liver) vs control, PP#, total hip arthroplasty


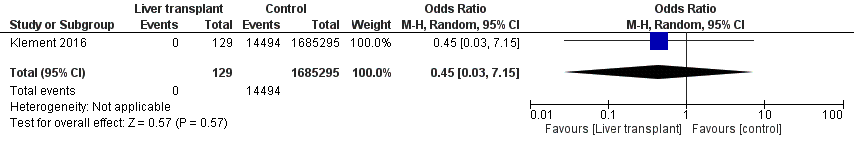


Solid organ transplant (liver) vs control, PP#, total knee arthroplasty


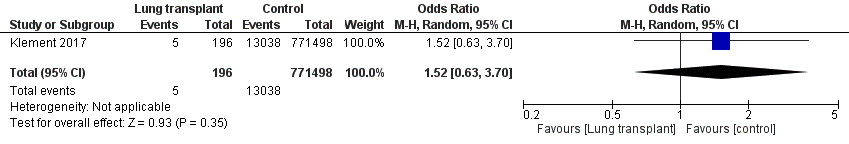


Solid organ transplant (lung) vs control, PP#, total hip arthroplasty


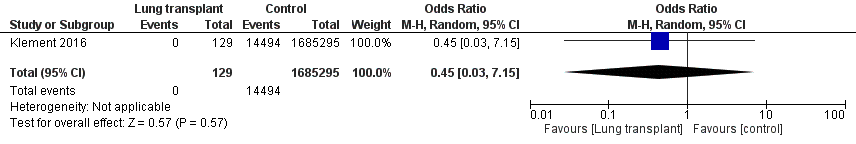


Solid organ transplant (lung) vs control, PP#, total knee arthroplasty


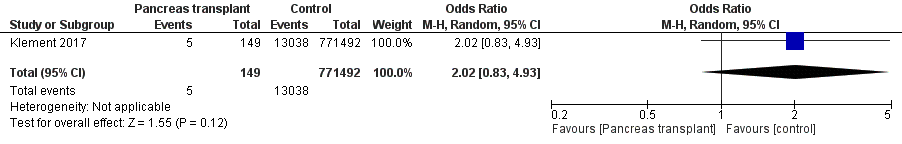


Solid organ transplant (pancreas) vs control, PP#, total hip arthroplasty


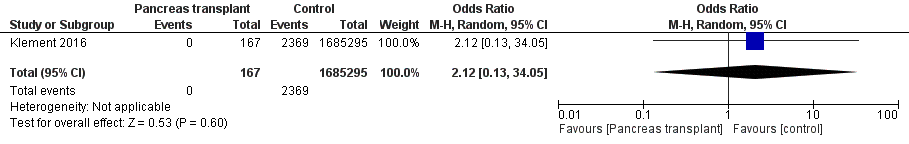


Solid organ transplant (pancreas) vs control, PP#, total knee arthroplasty


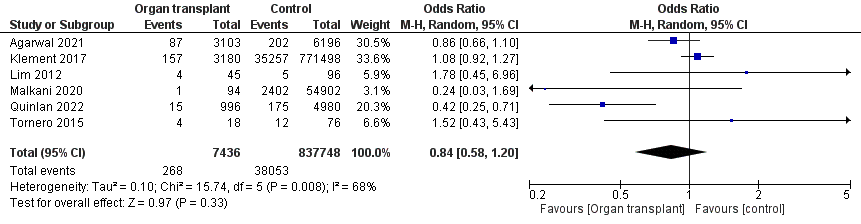


Solid organ transplant (all types) vs control, revision, total hip arthroplasty


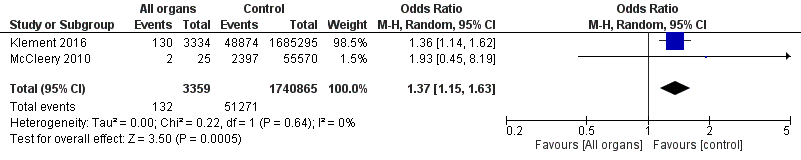


Solid organ transplant (all types) vs control, revision, total knee arthroplasty


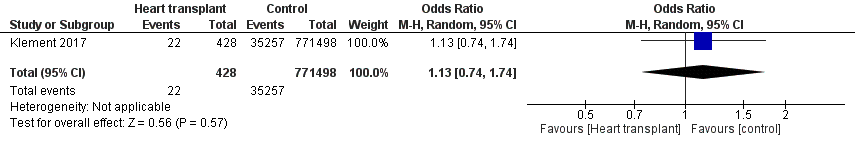


Solid organ transplant (heart) vs control, revision, total hip arthroplasty


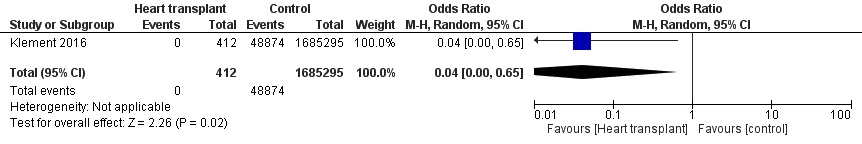


Solid organ transplant (heart) vs control, revision, total knee arthroplasty


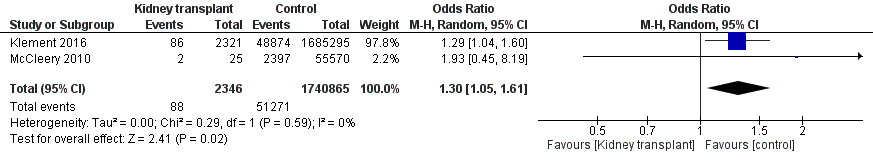


Solid organ transplant (kidney) vs control, revision, total hip arthroplasty


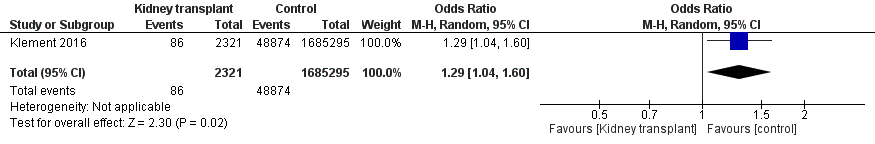


Solid organ transplant (kidney) vs control, revision, total knee arthroplasty


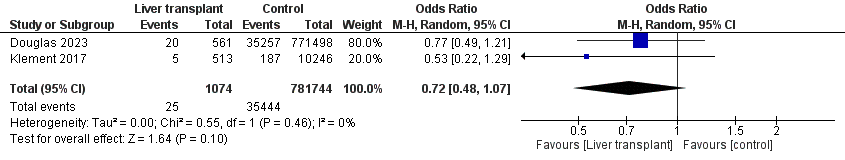


Solid organ transplant (liver) vs control, revision, total hip arthroplasty


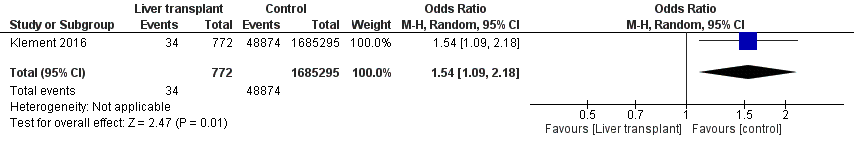


Solid organ transplant (liver) vs control, revision, total knee arthroplasty


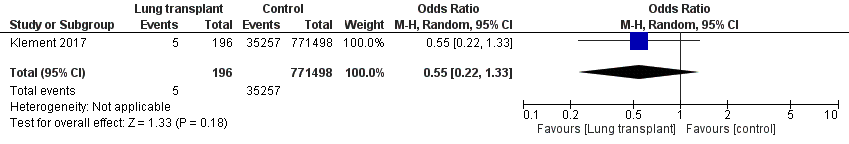


Solid organ transplant (lung) vs control, revision, total hip arthroplasty


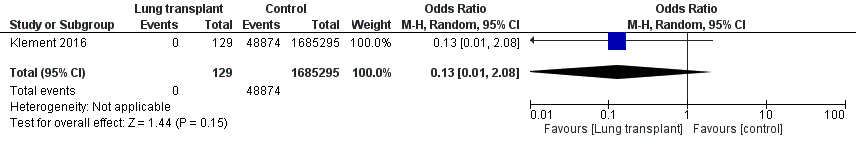


Solid organ transplant (lung) vs control, revision, total knee arthroplasty


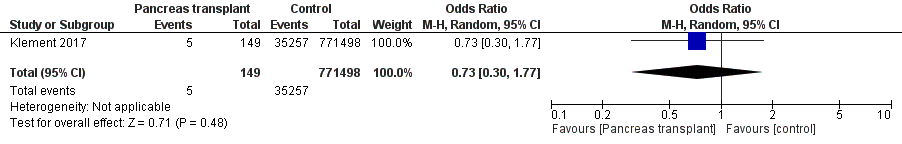


Solid organ transplant (pancreas) vs control, revision, total hip arthroplasty


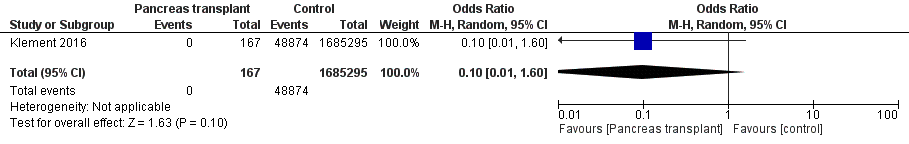


Solid organ transplant (pancreas) vs control, revision, total knee arthroplasty


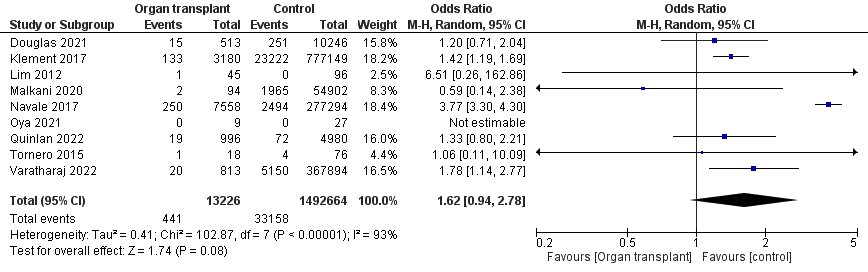


Solid organ transplant (all types) vs control, dislocation, total hip arthroplasty


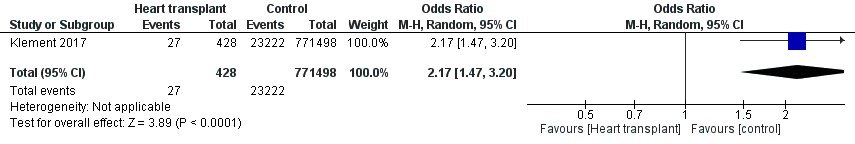


Solid organ transplant (heart) vs control, dislocation, total hip arthroplasty


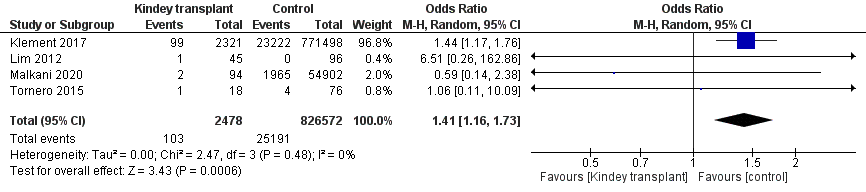


Solid organ transplant (kidney) vs control, dislocation, total hip arthroplasty


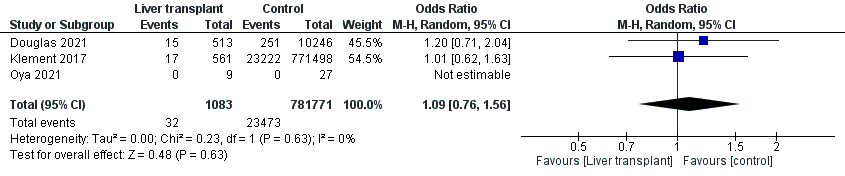


Solid organ transplant (liver) vs control, dislocation, total hip arthroplasty


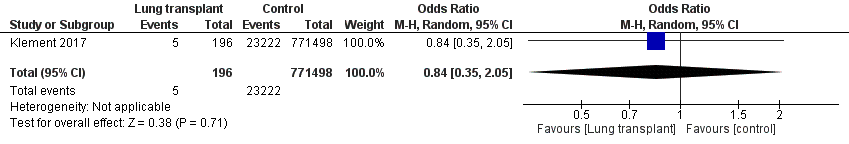


Solid organ transplant (lung) vs control, dislocation, total hip arthroplasty


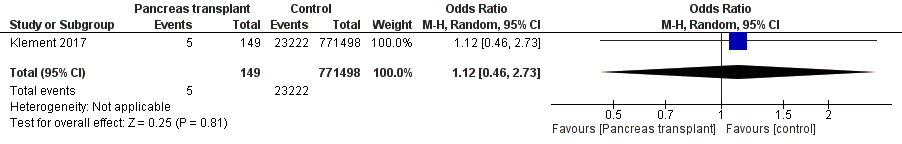


Solid organ transplant (pancreas) vs control, dislocation, total hip arthroplasty
